# Convention of Western Medical Schools

**Published:** 1849-01

**Authors:** 


### ARTICLE III.

### CONVENTION OF WESTERN MEDICAL SCHOOLS.

The Western Lancet in noticing the proposition for a convention of Western Medical Schools, made in the Ohio Medical and Surgical Journal approves of the suggestion and then uses the following language, p. 385.

Our cotemporary seems to present the proposition for a convention as something new; we would beg to inform him, however, that we published a similar proposal in the Lancet for January last. To that proposition not a single school responded.

Now if there is any particular merit in priority in reference to this convention we may claim to be the first delegate appointed to attend it. We would correct the Lancet in reference to there being no response to the call made last January, in which it was requested that notice should be sent to the Editor of that Journal. When appointed a delegate with our Colleague Prof. Fitch, by the faculty of Rush Medical College to attend it, we notified Prof. Harrison, one of the Editors of the Lancet, of the fact, and have his reply which states that the project had failed.

We suppose Dr. Lawson wrote the recent notice, and was not aware of these facts. E.

